# “The message is the manner”: Patterns of influence in communicating pap screening in North-Central Nigeria

**DOI:** 10.1016/j.qrmh.2025.100004

**Published:** 2025-03-25

**Authors:** Nancin Dadem

**Affiliations:** College of Humanities and Sciences, Coker University, United States

**Keywords:** Cervical cancer, Pap screening, PEN-3, Interpersonal and group communication, Nigeria

## Abstract

Effective strategies to closing the knowledge gap on cervical cancer and pap screening are needed to increase screening rates and create a greater demand for services in Nigeria. Using the PEN-3 Cultural Model, this paper facilitates understanding of cultural influences on women’s decision to screen for cervical cancer. The study draws on qualitative interviewing to explore women’s experiences with pap testing and to describe how cervical cancer prevention is perceived, communicated, and utilized in the population. Data consisted of interviews generated from a purposeful sample of 63 adult female participants. An iterative approach was used to abductively synthesize data to identify themes. Analyses produced three themes: knowledge and relational motivation, risk and barrier perceptions, and getting the word out. Findings highlight the potential in applying culturally centered approaches to promoting cervical cancer prevention in underserved populations.

## Introduction

In contrast to gains in cervical cancer prevention efforts achieved in high income countries attributable to early detection with advanced screening methods (Ba et al., 2021), an increase in invasive cervical cancer at advanced stages has been recorded in Nigeria due to late presentation and diagnosis (Ba et al., 2021, Musa et al., 2016). Records indicated 14 % pap screening rate in Nigerian women (World Health Organization, 2021). Studies reported poor knowledge of cervical cancer prevention in Nigeria (Balogun and Omotade, 2018, Olubodun et al., 2019), and similar concerns have been articulated in cervical cancer studies across Africa south of the Sahara (Black et al., 2019; Wilson, 2021). A significant approach to tackling the problem is closing the existing knowledge gap. However, literature suggests that increasing knowledge alone rarely instigates behavior change (Wright et al., 2008).

Because behavior is intertwined with culture, Wright et al. (2008) warned that behavior change is not facilitated by simply providing information about people’s risk to a health problem, but by understanding individual health and health risk perceptions and the critical role of cultural and psychosocial influences. Therefore, factors beyond individual cognitive abilities need to be investigated. Accordingly, this study examined the cultural influences that shaped women’s decision to screen for cervical cancer.

Importantly, the dearth of cervical cancer knowledge and pap screening in Nigeria calls for identifying effective communication strategies for urgent intervention to mitigate the burden of the disease. Sensitive topics, such as women’s reproductive health, require cultural sensitivity to achieve buy-in of screening from target populations (Krieger et al., 2013). Moreover, close-knit communities highly regard family and interpersonal communication, with health information much more valued from family health decision makers and close relationship networks than from public media. (Krieger et al., 2013). Thus, owing to the benefits of relational communication as a precursor to healthy living (Goldsmith & Albrecht, 2011), this study explored women’s experiences with pap screening to reveal cultural influences on their decision-making and to identify effective communicative attributes that may increase knowledge and pap testing in Nigeria’s North-Central region.

### Cervical cancer screening in Nigeria

Social determinants of health, such as lack of knowledge about disease symptoms and benefits of pap screening (Abugu and Nwagu, 2021, Okunowo et al., 2018), cost of pap screening (Abugu and Nwagu, 2021, Chigozie et al., 2022), and ignorance of screening locations (Abiodun et al., 2013, Abugu and Nwagu, 2021) undermine screening. Other factors include low-risk perception and susceptibility to cervical cancer (Abugu and Nwagu, 2021, Attah et al., 2019, Chigozie et al., 2022) and fear of a positive test result (Abugu and Nwagu, 2021, Attah et al., 2019). Okunowo et al. (2018) specified the lack of awareness about disease symptoms and non-recommendation of pap tests by healthcare providers, even among patients aware of pap testing, as foremost reasons for women not having had one. Conversely, doctors and nurses’ recommendation of pap tests and the fear of developing cervical cancer were main determinants and predictors of screening. Searching for solutions, researchers emphasized the critical role of healthcare providers in providing regular education to women attending clinics (Attah et al., 2019, Okunowo et al., 2018) and recommending pap testing to improve screening rates (Okunowo et al., 2018). Abugu and Nwagu (2021) advocated for an individualized and targeted education approach based on evidence from their study finding that women were generally aware of cervical cancer and screening, but needed information on other important aspects of the disease such as the causes of cervical cancer and its signs and symptoms.

However, Dim et al. (2009) and Okunowo et al. (2018) distinguished between awareness (familiarity) and knowledge (facts and information) and cautioned that an increased awareness on cervical cancer and pap testing may not result in increased screening. The Dim et al. (2009) study revealed low utilization of screening (18 %), even among female medical practitioners in Enugu, Eastern Nigeria. Although the Dim et al. (2009) study dates 15 years back, it shares similar observation with the Attah et al. (2019) study in which authors reported lower screening amongst doctors (15 %) as against 32.5 % and 20 % amongst nurses and community health workers, respectively, in a hospital in Plateau State, North-Central Nigeria. This finding was especially disturbing considering that doctors had 97.5 % correct knowledge about pap testing for cervical cancer.

Okunowo et al. (2018) confirmed a high level of awareness about cervical cancer (78.5 %) in the study population in Lagos, Western Nigeria, yet knowledge regarding symptoms and risk factors of cervical cancer and uptake of pap testing (22.9 %) were poor. Similar findings of 78 % awareness of cervical cancer, but only 20 % screening participation were reported in immigrant and refugee populations from Somali and Mexican extraction in central Ohio, United States (Gebre et al., 2021).

A significant percentage of healthcare workers (51.7 %) and non-healthcare workers (46.5 %) believed they were not at risk for cervical cancer (Attah et al., 2019). Major reasons for low-risk perceptions across study respondents were centered on religious faith in divine protection summarized as “not my portion” (a Nigerian spiritual expression rejecting harm), no family history of cervical cancer, and not being promiscuous (Attah et al., 2019). While the majority in both healthcare and non-healthcare groups decided to screen for cervical cancer, 58 (24.2 %) of the healthcare personnel and 65 (18.3 %) of non-healthcare personnel declined because they believed that they did not have cervical cancer (50 %), believed in the religious assurance of good health (30 %), and/or feared being detected with the disease (20 %) (Attah et al., 2019). Likewise, in a study of a population of men, Chigozie et al. (2022) reported low-risk perception of HPV and cervical cancer (68 %) stemming from men’s disbelief of their family members’ susceptibility to cervical cancer. Respondents in the Abugu and Nwagu (2021) study expressed similar doubts of susceptibility to developing cervical cancer and being fearful of a positive result as reasons for not having had a test.

Additionally, Nkwonta et al. (2021) suggested that the association between the sexually transmitted human papillomavirus and cervical cancer contributes to cervical cancer stigma, leading to a likely reluctance to screening in Nigeria. Similarly, Chinese women found it difficult to accept the connection between sexual activity, HPV infection, and cervical cancer and indicated feelings of stigma and shame arising from positive HPV results detected during screening (Lee & Craft, 2002). In South Africa, women reported that cervical cancer was disgraceful because of its association with promiscuity and would rather not report symptoms (Wood et al., 1997).

Balogun and Omotade (2018) reported that participants perceived women’s promiscuity as the cause of cervical cancer in all 20 focus groups in a study in Ibadan, Western Nigeria; likewise, traditional healers and older men viewed women as “reservoirs of diseases,” as their bodies were thought to conceal sicknesses. Balogun and Omotade further emphasized cultural and religious expectations of chastity before marriage in the discussions of HPV vaccines for primary prevention in adolescents and respondents’ belief of cervical cancer being a curse and consequence of wrongdoing (i.e., sexual sin).

Taking religion into account, Abugu and Nwagu (2021) recommended the involvement of faith-based organizations in cervical cancer prevention programs knowing the influence of these institutions in the daily lives of Nigerians. This research and the studies summarized above all emphasize the immense value of exploring local, regional, and national cultural influences on women’s cervical cancer prevention behavior to identify effective strategies for intervention.

### Theoretical foundation

Theorists have long argued the need to position culture and culture-centered approaches in defining health problems and framing solutions (Airhihenbuwa, 1989, Airhihenbuwa, 1995, Dutta, 2016). Culture shapes people’s sense of reality and informs their beliefs, practices, and shared values, distinguishing what is expected from what is unacceptable in a society (Napier et al., 2017). A useful tool in understanding the impact of culture on health behavior is the PEN-3 model which emphasizes three key elements: 1) cultural identity (person, extended family, neighborhood); 2) relationships and expectations (perceptions, enablers, nurturers); and 3) cultural empowerment (positive, existential, negative) within a health context (Airhihenbuwa, 1989, Airhihenbuwa, 1995).

Cultural identity is said to be the entry point for sensemaking and intervention occurring at the individual level of person, extended family, and neighborhood (Airhihenbuwa, 1995, Iwelunmor et al., 2014). Thus, on one hand, health communication researchers link relationships and expectations to identify 1) health attitudes and perceptions, 2) structural resources such as health services that (dis)enable the utilization of services, and 3) influences of family in decision-making and management of health issues (Iwelunmor et al., 2014, Olufowote, 2021). On the other hand, researchers use the cultural empowerment domain to identify positive beliefs and practices that facilitate health and the negative beliefs and practices that serve as barriers (Iwelunmor et al., 2014, Olufowote, 2021). Thus, health behavior points us to the meanings of pap screening and cervical cancer as constituted and defined by culture in PEN-3. Accordingly, two questions guide this inquiry:

*RQ1*: What knowledge and experiences do women in North-Central Nigeria have about pap screening and cervical cancer?

*RQ2*: How do women in North-Central Nigeria decide whether to participate in pap screening?

## Methods

### Ethical approval

The Human Ethics Committee of the Institutional Review Board at Ohio University, Athens, Ohio, United States, and the National Health Research Ethics Committee of the Federal Ministry of Health, Abuja, Nigeria, approved this study. To protect participants’ confidentiality, data are not available. I assigned pseudonyms to participants.

### Sampling and recruitment

Following IRB approvals, I utilized purposeful sampling to recruit study participants aged 25 and above in accordance with WHO’s recommended age for commencing cervical cancer screening (World Health Organization, 2013). Four experienced interviewers in Nigeria solicited study participation through multiple media. Interviewers contacted potential participants by leveraging past research relationships in some participating communities at study locations. Women leaders known to interviewers were informed about the study and provided copies of study announcements to share with other women in their communities. Those interested in participating reached out to the interviewers through phone calls, and interviews were scheduled and conducted. Others communicated their interest in participating to women leaders, who in turn, informed the interviewers to reach out to potential participants and secure convenient dates and times. Third, women leaders directly mobilized women on behalf of the interviewers and arranged interview venues and times. Participants used either signatures or thumb prints on the consent forms as indication of intent to participate in the study. Interviewers conducted 63 individual, in-person interviews.

### Study sites

Interviewers carried out research interactions in Jos (Plateau State) and Abuja (Federal Capital Territory) in Nigeria’s North-Central region. I selected both locations principally for their availability of cervical cancer and HIV treatment programs where vulnerable women who stand greater risks of developing cervical cancer can access services. One significant aspect of my research goals is to understand what motivates the adoption of recommended health behaviors, especially where prevention is possible and available. Some of the largest HIV treatment programs in the country are situated in Jos and Abuja, including the United States President’s Emergency Plan for AIDS Relief (PEPFAR) supported program at the Jos University Teaching Hospital that serves as a hub for HIV treatment in the North-Central geopolitical zone (Kolawole et al., 2017). The Operation Stop Cervical Cancer (OSCC) unit in Jos began providing cervical cancer screening services to women in Jos, adjoining cities, and states across northern Nigeria in 2006 (Musa et al., 2020). In Abuja, the National Hospital and University of Abuja Teaching Hospital provide HIV and cervical cancer services. The availability of these cervical cancer screening programs, which are not obtainable in all the states of the country (Daniel et al., 2020), make Plateau and Abuja significant settings for this study.

To provide some geographic and demographic background, the North-Central region is made up of six federating states and Abuja (Constitution, 1999). The states are Benue, Kogi, Kwara, Nasarawa, Niger, and Plateau (Constitution, 1999). A collection of ethnic nationalities is found in the North-Central region which Nigerians refer to as “minorities” when compared with the three major groups (Hausa, Igbo, and Yoruba) in the country. The religious persuasions of the citizens within the region are Christianity, Islam, and Traditional Religion. The population of the North-Central region is approximately 30 million with a total area size of about 242,425 km^2^ (City Population, 2016). The heterogeneity of the region makes it interesting for understanding health behaviors and practices within complex settings to inform intervention.

### Research assistants

Four interviewers experienced in qualitative data collection and one expert transcriber assisted in collecting and processing data. Interviewers (two in Jos and two in Abuja) recruited participants, administered written informed consent, interviewed participants, audio-recorded interviews, and transmitted interview recordings.

Before the interviews, I explained the research study to the interviewers by going over data collection materials and the principles of ethical interviewing in a Zoom video call. Two interviewers and I participated in mock interviewing to model expected standards as I interviewed one of the interviewers as a role participant. This exercise was valuable for reinforcing probing skills. Furthermore, as the first five interviews were completed in each location, I listened to the recordings to identify gaps that could be strengthened in subsequent interviews. After listening to the interview recordings, I provided feedback to the interviewers to strengthen subsequent probing. Finally, I randomly listened to recorded interviews and compared them with corresponding transcripts to ensure accuracy in transcription.

### Data collection

Interviewers used a standard semi-structured interview protocol during interview sessions, but allowed for iteration, probing, and digression on relevant themes (Tracy, 2019). Interviewers began conversations by first explaining the purpose of the interview, obtaining written consent, and collecting socio-demographic data (age, religion, location of residence, education, occupation, and ethnicity) for meaningful insights into patterns of behavior. Interviews were conducted in English and Hausa and audio recorded with permission from participants. Audio recordings in Hausa were transcribed into English.

After the interviews were completed, interviewers encouraged respondents who had no knowledge of where to access pap testing services or were yet to screen to screen and provided written information on health facilities offering pap testing. This dimension is meaningful, considering the recommendation to view the research interaction as an intervention. Winfield (2022) challenged scholars to employ research as a type of activism and intervention—an avenue to share stories and connect Black women or minoritized groups to appropriate resources.

Interviews lasted between 40 and 60 min, and participants were compensated with $10 for their time. A total of 63 in-person interviews comprise study data, with 47 interviews conducted in English and 16 in Hausa. In Jos, 32 interviews were conducted in six locations, and in Abuja, 31 interviews were carried out in eight locations (Table 1). These locations are in proximate distance to healthcare facilities offering pap screening.

**Table 1:** Breakdown of study locations by site and number of participants.

Jos								
Angwan Rogo	Gyel	Mai Adiko	Tudun Wada	Lamingo	Kabong			Totals
6	6	5	6	4	5			32

Abuja								
Kuje	Gwagwalada	Garki	Dutsen Alhaji	Wuse	Mpape	Kubwa	Zuba	
5	5	5	3	3	7	2	1	31

### Characteristics of study participants

The mean age of participants was 42 years. Of the 63 participants, the majority 46 (73 %) had secondary school diplomas, associate degrees, and undergraduate degrees. A range of occupational roles was represented, such as teacher, civil servant, tailor, cleaner, and petty business trader. Forty-seven (75 %) participants identified as Christian, 11 (17 %) Muslims, and five (8 %) did not indicate their religion or had missing data.

### Data analysis

I analyzed data using a bottom-up/emic approach (Tracy, 2019). I utilized an iterative approach to abductively synthesize and analyze data to identify salient themes (Tracy, 2019) linking cultural influences and pap screening behavior. As Tracy detailed, an iterative approach to qualitative data involves moving back and forth using research questions, study data, and existing theories and scholarship throughout the process of data analysis. I began the analytic process with a complete reading of the interview transcripts. I assigned sections of text from interview transcripts to the categories to which they corresponded.

Borrowing from Tracy (2019), I observed primary-cycle coding, which is the first step in the process of sense making—from raw data to codes—by allocating descriptive words or phrases corresponding to emerging ideas and then developing themes. Throughout the coding process, I used in vivo codes and continually compared data relevant to each code to modify code definitions according to new data (i.e., the constant comparative method) (Tracy, 2019). Following my first cycle of coding analysis, I shifted to finer distinctions in a second-round process to identify emerging themes (Tracy, 2019). The first round of coding focused on in vivo codes that matched research questions on knowledge, perceptions, motivation, and obstacles such as “Information sources on cervical cancer and pap screening: clinic health talk, radio program, friend’s death, and colleague,” and “preferred communication strategies: language, religion, one-on-one, politeness, information, women groups, and social media.” While the second round of coding analysis collapsed the first-round of codes, grouping multiple codes with related ideas or similar patterns together into categories such as “knowledge and relational motivation” and “getting the word out,” thereby allowing themes to emerge from data.

### Trustworthiness of the research

#### Credibility

Through the values of self-reflexivity and thick rich descriptions, I immersed myself in the data to present rich descriptions and to draw conclusions. Being reflexive, I interrogated my positionality as I juggled through the process of data immersion and meaning making. I recognized that I was an “insider-outsider” (Smith, 2012)—an indigenous female from Nigeria’s North-Central region currently in a new setting which places me within multiple cultures. As such, I considered my background (views, personal experiences, implicit bias, and culture) that could influence the interpretations of data (Creswell and Poth, 2013, Tracy, 2019) and took steps to minimize bias by consistently referring to respondents’ narratives to establish and clarify meanings. I used the first-person voice to reveal my presence and influence in the process (Tracy, 2019). Plus, I used thick description by making choices on what illustrative quotes to include predicated upon the values of true representation—voices of the participants and interpretations that corresponded to participants’ points of view—attempting to perceive meaning without distortion (Van Maanen, 2011).

## Findings

Three broad themes revealed women’s experiences and cultural influences on their perceptions and decision to screen for cervical cancer: knowledge and relational motivation, risk and barrier perceptions, and getting the word out. I will elaborate on each theme and provide illustrative examples. Subthemes will be discussed within the last two themes.

### Knowledge and relational motivation

Most participants (80.1 %) were aware of cervical cancer and pap screening and had screened for the disease. Women’s awareness of cervical cancer and pap tests was concordant with their reports of having undergone pap testing in the past. They described cervical cancer as a type of cancer that affects “women”, “women’s private part” (vagina), “womb”, and “cervix”. However, three women indicated being unaware of what the test was about prior to testing because they were only instructed by providers to go for it. Those who never screened attributed it to being pregnant at the time when they became aware of the test, feeling nonchalant about testing, or not knowing where testing was done. About half of the women lacked knowledge of the causes and risk factors of cervical cancer, though they felt at-risk for the disease. They expressed their ignorance and took the opportunity to ask about the facts from interviewers. Mara, (35, Jos), for example, commented:

That is what I don’t really know. They always emphasize on women going for the screening, to always go for the screening. I also want to know the causes. I’m so eager to know because I will be able to tell someone […]. But aunty [interviewer], is lack of sex one of the causes of cervical cancer? Now, I lack sexual desires towards men, so I was thinking if cervical cancer can cause that.

Others mentioned a variety of causes ranging from sexual intercourse including oral sex, multiple sex partners, chronic vaginal infections, poor vaginal hygiene, and “toilet infection.”

Regarding participants’ sources of cervical cancer and pap screening information, women indicated multiple sources of interpersonal and group communication and public media. Their descriptions of interpersonal and group interactions included doctors’ recommendation, nurses’ health talks in the hospital, women’s meetings, a friend, family member, or colleague’s admonition, knowledge of someone suffering or had suffered from the disease, and door-to-door awareness campaigns. Public media sources consisted of social media, the Area Council Motor Park campaign,^1^ radio program, and television news. On getting cervical cancer information from women group meetings, Tani (47, Abuja) stated:

I don't know anything. But I went for one forum meeting like that [… one] woman [gave us a] health talk. But before I reached [got there] that day, she was almost done with her talk. But what I know I met [heard] was her talking about cervical cancer. That [it] is what is in the town now. That everybody should try and be doing [get pap tests regularly] tests every time to know your status. The woman gave [a] talk at every meeting. We have that meeting every two weeks and it was not even quite long.

Further analysis revealed women’s motivation to screen resulting from information and recommendation of pap screening by healthcare providers, information received from family and friends, and discussions at group meetings. For example, Sawaba (35, Abuja^2^) recalled how her late mother’s counsel motivated her to screen:

It was my late mother that advised me that she heard that they are doing [cervical] cancer tests, and according to her, it is very dangerous. [She said] we should go and do the test so that we’ll know our fate. I now went [with] her. Unfortunately […] she passed away. It was her support […] that was the best. She asked me to go, and I did.

Sharon (62, Jos) stated, “It is the information I got that helped me. I heard it [from the internet] but I was not motivated, I didn’t go. It was when the nurse spoke that I was pushed to go in for it”. Bethel (37, Abuja) described how her friend encouraged and motivated her to screen for cervical cancer:

The encouragement my friend gave to me. Because she helped me to realize my status [cervical cancer] and to know my stand. It was a friend that helped me. Just a friend, but she has been like a sister to me, because she has been helpful even in my health challenge. She has been the person advising me giving me hope and courage. What made me to take the decision then was that it [was] better for me to know it […and], I don’t need to be scared.

Palmatu (47, Abuja) recounted her experience:

On the 3rd of March, 2022 at [location…], we all went in one after the other and did it [pap test]. At first, when someone told me how the test process [of pap testing] was, I said I won’t do it, but some of the women encouraged me that it was a painless procedure. So, I went ahead and did the test, and when they told me the result, I was very happy.

Relatedly, as women recounted relational influences on their decision to screen, they also explained motivation from a psychological stance; i.e., the need to maintain “peace of mind” as well as avoid pain and death.

Participants described how support received directly from family and friends influenced them to screen for the disease. Information, encouragement, and monetary aid also proved instrumental in facilitating screening behavior. Many participants described receiving help by way of free pap testing in healthcare facilities and financial handouts from family members. Those who reported receiving cash support to get a pap test requested for it from family members to make up for cash shortages: “Someone assisted me. A family member. It’s just from a family member that I called to help me with a little cash to complete the one I have” (Lilian, 40, Abuja). “Yes. My elder brother. I told him that I don’t have money to run the test, so, he gave me” (Mara, 35, Jos). And Ariella (30, Abuja) explained:

I got it from my dad, though I just told him that I want to go for a checkup. You know as a lady; you cannot go and start explaining to them like that [….] I said I want to go and check myself. I didn’t know how to start explaining to him because he is not all that educated to understand. So, I just said I just want to go for a checkup.

Likewise, two women reported screening because of group proactiveness and influence. These women emulated other group members and acted upon the pap screening instruction shared. Promise (age not indicated, Abuja) narrated her experience this way:

I went for the test because everybody was doing it […] They call us when there is anything like this because I’m in that [health] support group. So, anything that happens, they will just call us to come. They encouraged us to do the test. They called me on the phone. They called my name [to] come to [name of hospital].

Notably, findings revealed that cervical cancer prevention efforts can leverage existing health support groups. Data demonstrate that women’s actions were influenced by nurturers and enablers in PEN-3 as both relationship and expectations (family and group relations and provider-patient relationships) and the cultural empowerment domain (free testing by facilities and existential health support group) overlapped in facilitating pap screening. Equally, the lack of information on causes of cervical cancer and screening locations typifies negative enablers within the cultural empowerment domain.

### Risk and barrier perceptions

#### Risk perceptions to cervical cancer

Participants perceived a high risk of acquiring cervical cancer. All but one viewed the disease as serious, of which early detection was considered a key to treatment. Participants described susceptibility based on the female external genitalia as they explained that the vagina was open and vulnerable to infections. They pointed to poor vaginal hygiene such as picking up infections from public toilets, using chemicals to wash the vagina, prolonging the use of underwear, not bathing up to three times during menstruation, and washing the vagina with dirty hands. Women also perceived cervical cancer as serious because of the “seriousness of anything cancer,” the rising number of cervical cancer deaths in the country, and the “hidden” (not easily observable) nature of the infection in its early stages. Perceptions were split between a general fear of cancer/potential death, and cervical cancer infection/death trends—i.e., knowledge of someone who had died from the disease and information received from other sources such as “medical breaking news.”

Most significant concerns among participants were centered on cervical cancer affecting internal organs, the possibility of a prolonged HPV infection before the manifestation of physical symptoms, and disease symptoms manifesting as some other health problems. For instance, Leah (44, Jos) shared concerns on women misjudging cervical cancer symptoms and consequently resorting to self-treatment, illustrating interrelated problems of self-diagnosis and self-medication embedded in the cultural patterns of the Nigerian society:

Many of them do not have the information […]. They are ignorant [about pap screening]. So, when they start feeling such a thing [symptoms], most of them resort to traditional herbs which are deadly […]. Women that have cancer tend to look at it as ancestral sickness that can be treated with herbs. So, I think they need awareness.

### Perceived barriers to pap screening

While all but 12 women had had a pap screening, they expressed concern over a variety of barriers to screening. Challenges included 1) the lack of information on cervical cancer, pap screening, and screening locations, 2) cultural and religious beliefs and expectations, 3) the cost of pap screening, 4) fear of a positive cervical cancer diagnosis, and 5) limited access to health information in rural locations.

#### Knowledge gap

Participants described gaps in knowledge about disease and symptoms, pap screening, and facilities offering testing. They communicated concern on poor knowledge and awareness about cervical cancer and prevention in both urban and rural settings, affirming findings by Balogun and Omotade (2018) and Olubodun et al. (2019) on the huge knowledge gap in Nigeria. For example, “A lot of people are not really aware of it yet, and a lot of people have not really done it” (Bela, 40, Abuja). And

The challenge is, if I know I will go, but if I don’t have the information, where can I go? I can’t. When you tell them [women] to go for screening, they will be asking you how am I going to do it? How much is it going to cost? Which place and which place will I go for this screening? How are they going to take it [conduct the procedure]? You understand? So, they have some reasons that will make them not comply with it. (Tani, 47, Abuja)

Tapmwa (31, Jos) explained not being married and fearing a positive diagnosis as reasons for not having had a pap test, but ultimately attributing her lack of testing to lack of knowledge about the disease, of which relevant knowledge might have helped to dispel her fears:

Well, I’m not married. I’m single, and I don’t think I have an infection that should lead to that. That’s what I think. Well, the truth is, sometimes we shy away from it. That consciousness of going to check there, but there’s that something that what if you check, you know, I don’t think I have the energy to face that […]. [But] if I had an in depth understanding of it, maybe I won’t be this scared to go and check. Well, a lot of people are suffering from it these days; so, I think it’s a pointer for every woman, especially me, to have a screening. It’s better for me to know and start treatment than to let it become something that will become a problem for me and my family.

#### Cultural and religious beliefs and expectations

Participants discussed women’s possible reluctance of admitting to being at risk of cervical cancer and the rejection of pap screening due to faith in divine protection, discomfort from insertion of screening tools, shyness from exposing their vaginas during pap procedure, and cultural preference of female providers. Women typically yield to male providers during childbirth, but find it uncomfortable “opening up” to them for other reasons such as pap screening:

I have been to another screening, but not that I planned to go for another screening. I was opportune again [had another chance to undergo a pap test] in the health center here in [name of facility] where another free one was done, and I think that I took almost every woman that was around me that day. But I don’t know. Only two of us succeeded in doing it. Others ran away. Like one of the women that ran away was telling me, “Ah, just like that, I will just go and lie down? They will chook [insert] things inside me; how do you [allow that?]. Ah no oh, I cannot allow them [….]” [But] then, there was the presence of a male nurse [….] Some women would not want a man to touch them in their private parts. (Pamela, 38, Abuja)

Most of us northerners, especially the Muslim folks, when a Muslim woman informs her husband about the method employed to carry out this test, I bet you, almost all the men will not allow that, but few may allow their wives. [So], create awareness before they go for the test. The health workers should educate them [men] extensively to know that the test is harmless and is of benefit to them […]. Send messages and fully educate them on cancer until they’re well enlightened to send their wives to do the test. (Palmatu, 47, Abuja)

And Angela (31, Jos) lamented about the religious perceptions of sickness held by some. She extended the discussion to include issues such as C-sections, signaling that “it is not my portion syndrome” (a faith declaration and belief in divine protection), is a barrier to addressing health problems. Pamela (38, Abuja) shed light on such deterring religious perceptions:

You know, there are people that no matter what you say, they will say, “God forbid I’m not a victim of cancer, God forbid! In fact, what will bring cancer into my body since I didn’t offend God? So, God will not allow cancer to get to me.”

The interpretation of cervical cancer to be spiritual punishment from sin, denial of being at risk to cervical cancer on religious beliefs in divine health and protection from harm, and shyness from “opening up” during pap examination, reveal negative enablers in PEN-3. Yet, findings reinforce the need for cultural sensitivity towards women’s preferences of providers and presents opportunities to develop educational content addressing deterring themes.

#### Cost of screening

Participants indicated cost as a perceived barrier especially amidst economic hardship and cost ranging from 6000, 8000, 10,000, 12,000, 15,000, and 20,000 (+) naira in Abuja. Women in Jos indicated 500 naira for pap screening and suggested that the cost of screening that could presumably encourage women to test should range between 500 and 2000 naira. The currency equivalence at the time of study was about 400 naira to one US dollar. Unsurprisingly, women in Abuja reported higher costs of pap screening due to the high cost of living in Abuja compared to their Jos counterparts. Though women benefited from free and inexpensive clinics which is a positive systemic enabler in PEN-3, the high cost of screening conversely poses as a negative enabler in the model.

Women detailed their views regarding shared responsibility between government and families, suggesting that the government should help create public awareness about the disease and reduce the cost of pap screening to increase affordability. Others looked to sponsors, possibly nongovernmental agencies, in providing free testing, for example:

I think we need sponsors, those that render help for women to be able to do this test. Because in our society today, most people are thinking of what to eat, not to pay money for a test. (Loveth, 36, Abuja)

Furthermore, participants pointed to the need for providing more screening services specifically in private hospitals which are better known for shorter clinic turn-around times in comparison to government facilities known for “delay” and “waste of time.”

#### Fear of a positive cervical cancer diagnosis, pain, and stigma

Participants expressed fears of positive results and pain from the testing procedure. Angela (31, Jos), for instance, explained that, “The greatest is fear. Just like my cousin would say, she’ll say that what you don't know does not kill you, but that’s a lie, oh! Sometimes what you don't know kills you and even faster.” Another participant agreed: “You know from the beginning when you see those tools coming out, you will think that they [want to] remove your cervix” (Asenath, 43, Abuja).

Six participants mentioned perceived stigma as a barrier. As Francesca (48, Jos) stated, “[Stigma] is also a problem, being afraid of what some people will say.” Pointing to heredity and the need for medical background investigation before marriage in another fearful scenario, Diana (43, Abuja) described “stigma by association” in which others share the consequence of relating with a stigmatized person (Phillips et al., 2012). She described how a woman’s family history of cervical cancer can potentially dissuade men from marrying into that family.

Nonetheless, Sharon (62, Jos) might have provided some insight into why real stigma, i.e., actual discriminatory behaviors, was not put forward as a deterrent and stated how stigma would eventually evolve when responding to the question about community members’ impression of those who screen for cervical cancer and their treatment of those living with cervical cancer:

You know, most people don’t know that cervical cancer can be transmitted sexually. How do they really treat them? I don’t know. I don’t know. I have not seen how these women are being treated. I think it is now that people are having that knowledge. They are beginning to have the knowledge that it is transmitted through sexual intercourse. So, I think with time, they will receive that treatment [stigmatization].

Sharon’s submission highlights the risk of future social stigma for those diagnosed with cervical cancer, as knowledge of its sexual transmission increases.

Simbema (32, Jos) described her fear of stigma in pap screening from her status as a single woman in a culture that expects sexual purity from the unmarried. This expectation can be classified as a negative aspect of the cultural empowerment domain in PEN-3 that hinders pap screening:

Like I said, for example, once I am sexually active…automatically, they’ll feel I’m wayward. That’s why. Because they’ll feel if I am not sexually active, I don’t even need to go for this test because one of the criteria [is], have you been sexually active and all that? So, I feel it’s a very big stigma.

Yet, Abilene (47, Abuja) related the discussion of fear and stigma to HIV, stating that stigma is the reason most women prefer to die in ignorance of their disease. Overall, fear of death, stigma, or pain portends difficulty for women who might face death, manage their illness, and sometimes, society’s judgment of them. Each of these descriptions of real and perceived fear help in explaining reluctance and avoidance and reveal cultural expectations within the relationships and expectations domain in PEN-3 that discourage screening.

#### Rural locations

Women drew attention to the plight of other women in rural areas who have worse access to health information compared to cities. This structural barrier and inequity reflect a negative enabler in PEN-3 needing intervention. Women believed that information was generally lacking in the population, and more so, in rural communities. Leah (44, Jos) summed up her concern this way: “Many are ignorant, that's the truth, especially the rural areas […]. Rural women hardly get the information we are privileged to get in the city. They say information is power. Information is the key.” Urban women’s concern about poor knowledge of cervical cancer and pap screening in rural locations spotlights “place” as a social determinant of health. While I am cautious that respondents may be overstating their perceptions on rural locations lacking cervical cancer information since rural women were not interviewed in this study, participants believed that closing the knowledge gap in both urban and rural areas would drive screening rates upward.

As demonstrated, both real and perceived barriers to pap screening reveal negative cultural empowerment in PEN-3: lack of relevant knowledge; perceived limited cervical cancer information in rural locations; unaffordable cost of testing; existential attitude towards self-diagnosis and self-medication; fear of a positive diagnosis; religious beliefs in divine health and healing; and cultural expectations of chastity before marriage disincentivize pap screening. However, the suggestion of sensitizing northern men about cervical cancer and the importance of pap screening to gain acceptance and buy-in from them is culturally relevant to improving pap screening. It is equally important to explore urban womens’ perception about limited cervical cancer information in rural areas for effective intervention.

### Getting the word out

Women identified multiple channels of communication for increasing knowledge of cervical cancer and pap screening regarding interpersonal, group, and public media approaches. They specified interpersonal communication by 1) word-of-mouth through one-on-one interaction among women and between healthcare providers and women patients, 2) organized lectures and talks in social/community women group meetings and houses of religious worship, and 3) the use of both traditional and social media. They identified the hospital setting as a natural location for providers to share information with women, as Asenath (43, Abuja) affirmed: “[I]t is through information you can reach them [women], as most of us come to the hospital for pediatric, antenatal, and family planning.” Women’s emphasis on the role of providers in detailing information on cervical cancer and pap screening to women corroborated their preference of healthcare providers as the most trusted source of health information. On one-on-one interaction among women, Palmatu (47, Abuja) recounted the advice given to her by a healthcare provider to share information on pap screening with other women in her community:

That day, the nurse told us after this screening that we should keep advising women in our communities to come for the screening. It is important for women to know their status because early detection is curable. This was the advice given by the health worker.

Significantly, participants articulated the importance of women who have screened using themselves as potential examples to convince other women to screen. Some participants demonstrated how they intend to speak to convince others, while others reported attempts at “convincing” and served as linkage to health facilities offering pap screening. They took up these responsibilities by themselves or at the suggestion of healthcare providers who depended on them, asking that they advised others. These quotes capture women’s sense of responsibility to other women:

[I can say], my fellow women, this is your sister speaking reliably […]. I have gone through cervical screening, and this thing is real [cervical cancer], and I’m free [negative]. So, I want everybody to come out at least to screen yourselves to know [so that you can] treat yourselves earlier before it will go to the extent that it cannot be treated. (Asenath, 43, Abuja)

Anyone that has done the screening before should tell and encourage other women to know and go for the screening. If someone is not knowledgeable about something, it is difficult to convince them to do it, but when they are aware that you did the test, it will make them want to do it. (Abilene, 47, Abuja)

Some of them may not really believe it. So, I think some of us that know and have [done it] can pass information about it to educate them more and to tell them, to convince them to come and do it. (Atara, 47, Abuja)

Even this study’s interviewing sessions became impetus for inviting other women to screen for the disease. Adah (54, Abuja) said: “Like now, as you told me now [interview], I can tell somebody, I can tell my friend somebody interviewed me on this. Oh, and it is something good that we should know our status.”

Similarly, the suggestion of involving faith leaders and places of religious worship as sources of support further reveals nurturers and positive existential cultural empowerment in PEN-3. Atara (47, Abuja) states this view on parish nursing:

Some people, you know, some believe in faith that God will do it [heal]. They believe that they will pray, and God will do it, and they will be healed even when they have it [cervical cancer]. So, from the church, even our pastors can help us, tell us about health and to do it [pap screening].

Participants identified nurturers as people they believed women would be comfortable listening to. They suggested that cervical cancer information can be shared in creative and convincing ways, such as using drama and language that can be generally understood within communities. The following example captures this sentiment:

In churches. Like I said, religion plays a major role in Nigeria. It might be through drama. Like in church, you could use special programs, maybe one of the women’s fellowships. The pastor's wife can just invite somebody that is knowledgeable, medical personnel. And then when he or she comes to give them a talk […] they might be more comfortable with a woman as in a woman-to-woman kind of thing. It can be done through drama, medical outreach to gather the people, it might be through speaking the language they understand. (Angela, age 31, Jos)

Women also suggested using traditional and social media to reach women with information on cervical cancer, the need to screen for the disease, and locations where pap screenings are offered. These channels would be for the purposes of announcement, sensitization, and mobilization for screening. Sharon (62, Jos), for example, recommended that “government through the media, radios, [and] televisions should sensitize people, women, concerning pap’s smear.”

Although women proposed the use of social media, they acknowledged the unequal access to digital telecommunication resources and suggested the use of social groups and networks to best meet the needs of the majority. Hajiya (46, Abuja) explained:

[Promote the information] on social media, but then, it’s not everyone that has an android phone. So, such information can be passed during meetings, in ceremonies, or campaigns announcing the effects of this disease and where to do it and all that.

Overall, with Abuja’s higher digital technological coverage and opportunities, it was unsurprising that more respondents (n = 10) in Abuja suggested using social media for creating cervical cancer awareness than in Jos (n = 6). These preferred communication styles and norms characterize the positive nurturers and enablers in the relationships and expectations domain at the interpersonal level and the existential enablers within the cultural empowerment domain at the collective level in PEN-3.

#### Informative messages

When it comes to content of information that would be valuable in informing and influencing women to screen, participants favored cervical cancer and pap screening messages that specified the importance of pap screening and early detection, screening procedure, information on causes of the disease and preventive behaviors, and screening locations. As Hajiya (46, Abuja) stated, “They should educate me on how this disease is affecting women and how I should take care of my health, [the] effect of the disease, and all that, and how to prevent myself from this disease.” Relatedly, Palmatu (47, Abuja) stressed the need for explaining to women that screening does not imply death when she advised: “You should fully educate them on the disease and make them understand that doing the screening is not a death sentence.” Mara (35, Jos), however, cautioned against healthcare providers’ emphasis on screening for cervical cancer without providing essential information on the causes of the disease and linked the importance of knowing such information to her empowerment and duty to telling someone about it: “They [providers] always emphasize on women going for the screening, to always go for the screening. I also want to know the causes. I’m so eager to know because I will be able to tell someone.” Again, this sense of responsibility towards other women articulated by participants specifies the cultural ways in which information is shared in the population, giving credence to the positive influence of social networks as stipulated in PEN-3.

#### Appropriate language and polite communication

When participants were asked about the treatment that they had received from healthcare providers during pap screening and their preference on how to be treated when undergoing subsequent procedures, women reported being treated exceptionally well in glowing and repetitive terms such as: “very, very nice,” “very wonderful,” “nice people,” “encouraging,” “very good manner,” and “treatment was very normal.” They described how persuasive providers spoke, using encouraging words to help them relax during insertion of screening tools, and delivered understandable lectures on the topic. Lilian (40, Abuja), for example, relayed some surprise with how well her procedure went as she described the “wonderful,” “great,” and “fantastic” experience she had, which in her words, made her feel like a family member. Gimbiya (45, Jos) recounted, “She [provider] spoke gently, and you will understand what she was saying. She gave a beautiful lecture, and it was very clear.”

On participants’ preference regarding how to be treated by providers, they emphasized that the language of communication with women should be one that is understood by them and relayed in a respectful manner and tone. The following examples demonstrate their perspectives: “The message is the manner as I said earlier. So, when you use a good manner to tell someone about it, that person will understand” (Eko, 40, Abuja) and “To treat us good, all the patients that will be going there. If they have manners of approach to the patients, at least, there will be understanding between the patients and the nurses there” (Tamara, 44, Jos).

Other women expressed similar sentiments about the tone of speech and mindfulness in the use of testing tools, for instance: “[By] using kind words and the nurses should be understanding that this is our body, we have feelings, and be tender inserting all those tools into someone” (Pamela, 38, Abuja). Angela (31, Jos) highlighted the importance of explaining cervical cancer and pap screening in the language women understood. She described how the knowledge gained by women can create a chain of promoters who would reach one woman after another, thereby spreading the information quickly.

On the other hand, despite many reports by women on being treated respectfully by healthcare providers who took their pap samples, Mara (35, Jos) described poor handling. She lamented how angry she was and still is at her treatment and stated how discouraging it was to return for subsequent screening:

I told you she spanked me that day, right? Yes, “Open, open your legs.” She wasn’t happy that I wasn’t opening my legs, and she hit me. I got angry too, and I said to myself, I won’t come back again for you to even think of hitting me again […].

Returning for future pap screenings thus depended on previous positive encounters, highlighting the role of provider communication competence in supporting and sustaining health behavior over time. Given substantial connection between providers’ communication competence and health and behavioral outcomes as established in previous research (Delaney and Singleton, 2020, Street, 2008), women in this study highlighted attributes of provider communicative actions that facilitated trust and the positive behavioral outcome of screening.

## Discussion

This study provides insights into cultural influences on women’s pap screening behavior among the study population in North-Central Nigeria. The study found that participants’ decisions to screen was primarily motivated by provider-patient relationships and family and social relationships identified as nurturers and enablers as suggested by the PEN-3 cultural model (Airhihenbuwa, 1989, Airhihenbuwa, 1995). Healthcare providers recommended pap screening, provided information on the disease including free testing opportunities for financial relief to women, and communicated trust, encouragement, and politeness in an engaging manner. Findings affirm researchers’ emphasis on the role of healthcare providers in facilitating the cause of pap screening as evidenced in extant literature (Abugu and Nwagu, 2021, Attah et al., 2019, Okunowo et al., 2018). While previous research (Abugu and Nwagu, 2021, Balogun and Omotade, 2018, Nkwonta et al., 2021) supports engaging culturally relevant nurturers like religious leaders, men, and community leaders, it overlooks the crucial role of women as agents for information sharing.

In this study, I specifically highlight the potential role of women in cervical cancer prevention efforts*.* Women’s articulation of their role in convincing others indicated a strong sense of responsibility. Women with knowledge or experience with cervical cancer and screening assumed the role of informants and persuaders for pap screening as they worked to convince others to screen for the disease by sharing their knowledge and experience of pap screening; offering encouragement to those hesitant to screen; and in some cases, serving as linkage to pap screening services. Examples from the sections on “Knowledge and Relational Motivation”, "Cultural and Religious Beliefs and Expectations", and “Getting the Word Out” above highlight women’s approach in not just “telling” but “convincing” other women to test for cervical cancer.

Next, whether as social or religious groups, the usefulness of group forums in improving the cause of cervical cancer and pap screening was established. Groups are viable interactive avenues for sharing information. The attributes and connectedness of social groups that facilitated women’s decision to screen for cervical cancer reflected groups’ proactiveness in sharing relevant information at meetings, maintaining close connections, and leveraging power to positively influence members. Hence, the two-way communication approach in interpersonal and group communication in increasing knowledge on cervical cancer and testing rates reinforces the need to apply culturally centered solutions to solving problems, rather than a singular, top-to-bottom style in mass media models.

Christian worship centers and neighborhood Arabic schools for women were noted to be useful places of influence and frequently visited among participants. Religious leaders are influential personalities and powerful stakeholders in the Nigerian state, and their involvement could viably strengthen cervical cancer education efforts. Due to Nigeria’s patriarchal culture, male involvement is crucial for women needing male approval and funding for pap screening. These culturally appropriate strategies for promoting pap testing reveal existing positive elements in the community that impact screening behavior as observed in PEN-3 and provide insights for organizing cervical cancer education interventions.

Key findings on women’s lack of knowledge on cervical cancer causes and symptoms, perceived barriers to pap screening such as fear of a positive test, and structural access problems such as cost and isolated geographic locations signify negative cultural empowerment. Likewise, the cultural and religious expectation of chastity conflicts with test-seeking behaviors in unmarried women and potentially weakens adoption of pap screening. These negative factors contend with positive influences, demonstrating the complexities of cultural and societal forces that disincentivize preventive behavior. Consequently, socio-cultural realities reinforce the utility of personal education for disease prevention and governments’ role in strengthening cervical cancer prevention. Based on findings regarding the high cost and poor knowledge of pap screening, government’s duty in increasing access to pap screening by informing the populace about the disease through radios and television for mass coverage, reducing the cost of screening, and increasing pap testing sites is required. Responding accordingly would require the Nigerian government’s funding commitment and bold action.

Also, given the findings, the nurturing and enabling influences of providers, family, friends, and groups play a pivotal role in increasing knowledge of cervical cancer and potential increase of existing pap screening services. In relation to health communication theory, cultural identity, relationships and expectation, and cultural empowerment all reflect the postulations of the PEN-3 model. As such, results illustrate the potential of PEN-3 for problem-prevention and intervention.

### Limitations

While this work offers significant insights for organizing cervical cancer prevention intervention efforts, it is limited in some ways. Most study participants had had a pap test in the past. Though the study was open to all women irrespective of their pap screening history, it can be assumed that women who had screened felt a greater sense of adequacy in responding to the study topic. However, understanding the perspectives of those who never tested for cervical cancer could reveal additional barriers and nuances to screening. In addition, all participants in this study were in Jos and Abuja, meaning that variations across other settings in North-Central Nigeria cannot be addressed. Similarly, only women living in urban areas in both study locations were reached in this study; therefore, results do not represent the experiences of rural women in the region. However, findings do not detract from the influence of relational networks that facilitated pap screening in this study population.

### Future direction

Since evidence from this study revealed positive influences of nurturers and existential enablers in facilitating pap screening, considerations for future research necessitate identifying and developing strategies to organize and integrate these key targets in communication research intervention plans. Such plans would incorporate women and community leaders and groups, alongside healthcare providers, in playing a leading role in educating and influencing women to screen for cervical cancer across the North-Central region and by extension, the Nigerian state. Similarly, understanding the difficult circumstances of rural women is imperative for better appreciation of the low pap screening problem and consequent intervention.

## Conclusion

Investigating women’s pap screening behavior through the cultural lens of PEN-3 provided valuable understanding of influences at the personal, familial, interpersonal, and collective levels. Relationships and expectations among cultural empowerment domains revealed positive, existential, and negative influences on pap screening behavior. Poor access to pap screening services including perceived high pap screening cost, inadequate screening facilities, and rural locations point to significant structural barriers viewed as negative enablers with the cultural empowerment domain in PEN-3. However, resources and influences through interpersonal and group communication in women’s social networks and positive provider-patient interactions enhanced women’s decision to screen for the disease.

Findings of this study point to relational networks as culturally relevant enablers in improving knowledge and increase of pap screening in North-Central Nigeria. If 1) women who have screened for cervical cancer inform other women and use themselves as exemplars, 2) healthcare providers share comprehensive details on cervical cancer and pap screening in languages women visiting the clinics understand in a friendly manner, and 3) groups regularly meet and provided opportunities to experts and influential figures to share cervical cancer information in creative ways, then efforts to improve pap screening rates would have increased chances of success. Along with these strategies, government’s concerted efforts are needed in closing infrastructural gaps and addressing information and mobilization needs.

This work offers insights for organizing cervical cancer prevention efforts focused on existing culturally relevant communication strategies in Nigeria. Findings from this study also offer a glimpse of what relational networks can significantly contribute to turning the tide of cervical cancer in Nigeria and accelerating WHO’s global target of eliminating cervical cancer as a public health threat by 2030 (World Health Organization, 2020). Benefits of this initiative for resource-limited countries such as Nigeria include a 42 % median reduction in cervical cancer incidence rates by 2045 and 97 % by 2120, thereby averting about 74 million new cases and 300,000 deaths by 2030; 14 million by 2070; and more than 62 million by 2120 (World Health Organization, 2020).

## Author statement

This study was funded by awards from Ohio University’s Council on Research, Scholarship, and Creative Activity; Graduate College and Graduate Student Senate; Scripps College of Communication Diversity Committee; and the School of Communication Studies’ Endah Agustiana and Rudi Sukandar International Travel Award. Portions of these findings were presented at the 2023 National Communication Association Convention, National Harbor, Maryland and Ohio University 2023 EXPO, Athens, Ohio, United States. I also published an opinion editorial in The Guardian, Nigeria. I have no conflict of interest to disclose. This study was conducted in Jos, Plateau State and Abuja, Federal Capital Territory, Nigeria. As author, I designed the study, interpreted data, and wrote the manuscript.

## CRediT authorship contribution statement

**Nancin Dadem:** Conceptualization, Data curation, Formal analysis, Funding acquisition, Investigation, Methodology, Project administration, Resources, Software, Supervision, Validation, Visualization, Writing – original draft, Writing – review & editing.

## Declaration of Competing Interest

The author declares the following financial interests/personal relationships which may be considered as potential competing interests: Nancin Dadem reports financial support was provided by Ohio University.

## Footnotes

1 Area councils are a form of local government administration in the Federal Capital Territory, Abuja. Campaigns organized by these administrations sometimes take place at public transportation hubs.

2 Ages and interview locations are provided in parentheses for each participant quoted.
